# D-Serine Is a Substrate for Neutral Amino Acid Transporters ASCT1/SLC1A4 and ASCT2/SLC1A5, and Is Transported by Both Subtypes in Rat Hippocampal Astrocyte Cultures

**DOI:** 10.1371/journal.pone.0156551

**Published:** 2016-06-07

**Authors:** Alan C. Foster, Jill Farnsworth, Genevieve E. Lind, Yong-Xin Li, Jia-Ying Yang, Van Dang, Mahmud Penjwini, Veena Viswanath, Ursula Staubli, Michael P. Kavanaugh

**Affiliations:** 1 Department of Biological Sciences, Allergan, Inc., 2525 Dupont Drive, Irvine, CA 92612, United States of America; 2 Center for Structural and Functional Neuroscience, The University of Montana, Missoula, MT 59812, United States of America; CNRS—Université Aix Marseille, FRANCE

## Abstract

N-methyl-D-aspartate (NMDA) receptors play critical roles in synaptic transmission and plasticity. Activation of NMDA receptors by synaptically released L-glutamate also requires occupancy of co-agonist binding sites in the tetrameric receptor by either glycine or D-serine. Although D-serine appears to be the predominant co-agonist at synaptic NMDA receptors, the transport mechanisms involved in D-serine homeostasis in brain are poorly understood. In this work we show that the SLC1 amino acid transporter family members SLC1A4 (ASCT1) and SLC1A5 (ASCT2) mediate homo- and hetero-exchange of D-serine with physiologically relevant kinetic parameters. In addition, the selectivity profile of D-serine uptake in cultured rat hippocampal astrocytes is consistent with uptake mediated by both ASCT1 and ASCT2. Together these data suggest that SLC1A4 (ASCT1) may represent an important route of Na-dependent D-serine flux in the brain that has the ability to regulate extracellular D-serine and thereby NMDA receptor activity.

## Introduction

D-serine has gained much attention as an endogenous co-agonist of the N-methyl-D-aspartate (NMDA) sub-type of glutamate receptors [1–4]. The dependence of NMDA receptor activation on occupation of the co-agonist site has led to the proposal that endogenous D-serine “tone” plays a key role in determining neuronal plasticity during development and in the adult brain [5,6]. The decline of D-serine with age has generated speculation that age-related deficiencies in cognitive ability may be linked to the lack of this co-agonist [7,8]. In schizophrenia, D-serine levels may also be reduced and contribute to the schizophrenic symptoms that have been attributed to a hypofunction of NMDA receptors [9]. Indeed, oral D-serine treatment has been shown to improve the negative and cognitive symptoms in schizophrenia [10]. Consequently, mechanisms in the central nervous system (CNS) that regulate D-serine are of great interest.

In the brain, D-serine is synthesized by the enzyme serine racemase (SR; [11]). Racemization of L-serine is the major source of D-serine in the brain, since knock-out of SR results in endogenous levels of D-serine that are <15% of control [12,13]. The primary localization of serine racemase has been controversial, but there is clear evidence that it is mainly present in adult neurons, and to a lesser extent in glial cells [14–17]. The predominantly glial enzyme D-amino acid oxidase (DAAO) is responsible for the catabolism of D-serine [18]. In the CNS, this enzyme is present primarily in the brain stem, cerebellum and spinal cord with reduced levels in higher brain regions [19]. Inhibitors of DAAO can elevate endogenous D-serine levels in these lower brain regions, but to a much smaller degree in the hippocampus, cerebral cortex and other forebrain areas, suggesting that DAAO has a less important role in regulating D-serine in higher centers [20].

Since D-serine is a polar amino acid that must be transported across cell membranes, the transporters that mediate cellular uptake and release of D-serine have been of great interest, as these are likely to play a critical role in regulating the extracellular and synaptic levels of D-serine that influence NMDA receptor activity. To date, the two major D-serine transporters in CNS tissues that have been kinetically characterized are the sodium-independent neutral amino acid exchanger asc-1 [21,22] and the sodium-dependent neutral amino acid exchanger, ASCT2 [23]. The heterodimeric asc-1 is formed by the product of two genes, SLC7A10 and SLC3A2, that combine to confer high affinity exchange of neutral L- and D-amino acids across cell membranes [21]. Release of D-serine from neurons via the sodium-independent asc-1 has been suggested to regulate synaptic NMDA receptor activity [24,25].

SLC710/SLC3A2 (asc-1) immunoreactivity is predominantly neuronal, and the sodium-independent transport of L- and D-serine in synaptosomes closely matches the properties of exogenously expressed asc-1 [22]. ASCT2 (SLC1A5) is expressed primarily outside the CNS [23]. Although ASCT2 expression in the brain is low, it has been associated with cultured astrocytes [26,27] and neurons [28,29] and in parenchymal cells [30] and retinal tissue [31]. ASCT1 (SLC1A4) is a related neutral amino acid transporter, and both ASCT1 and ASCT2 operate as exchangers whose substrates are small neutral amino acids such as serine, alanine and threonine. A specific physiological function of ASCT1 and ASCT2 has not been clear other than facilitation of amino acid exchange into cells for basic metabolic needs [32].

In the present experiments we evaluated the transport of both L- and D-serine in cultured astrocytes from rat brain and found that two components of sodium-dependent transport were present, consistent with functional ASCT1 and ASCT2. Moreover, D-serine was transported by both components, suggesting that D-serine is a substrate for both ASCT1 and ASCT2. This was confirmed in experiments where human ASCT1 or ASCT2 were heterologously expressed in HEK cells or in the *Xenopus* oocyte expression system. In addition, substrate differences between the two ASCT subtypes have provided selective tools that can be used to distinguish them. These results expand our knowledge of the transport systems that are responsible for D-serine regulation, and indicate an underappreciated role for ASCT1 in D-serine homeostasis.

## Materials and Methods

### Animals

All rodent experiments were approved by the Allergan IACUC and carried out in accordance with the National Institutes of Health guide for the care and use of laboratory animals. *Xenopus laevis* used in this study were treated in a manner to minimize suffering, and were anesthetized with tricaine prior to oocyte removal in accordance with NIH and University of Montana IACUC regulations (IACUC protocol approval 065-11MKBMED-122111)

### Cell Culture

#### Primary astrocyte cultures

Postnatal day 8 Wistar rats were anesthetized with isoflurane and decapitated. Hippocampi were removed rapidly under stereomicroscopic observation using sterile conditions, cut into 1 mm pieces, transferred to a tube with 0.7 mg/ml protease dissolved in PIPES solution (130 mM NaCl, 1 mM CaCl_2_, 5 mM KCl, 10 mM Pipes, 25 mM Glucose and 1 mM MgCl_2_, pH was adjusted to 7.4 with NaOH) and incubated at 33°C for 30 min with 100% oxygen bubbling into the solution. After removal of protease, the tissues were washed twice in culture medium (Neurobasal with B27 supplement (Invitrogen), with 2 mM L-glutamine and 10% fetal bovine serum), and gently triturated with ten to fifteen passes through the 0.78 mm opening of a tip of a P-1000 Pipetman. Cell suspensions were gravity-filtered through a 70 mm Nylon mesh (Falcon, Oxnard, CA) to remove large debris. Approximately 1–2 million cells were plated in a T75 flask (Corning, Corning, NY). Cultures were maintained at 36°C in a 5% CO_2_ incubator. After 24 hours the medium was removed and replaced with 14ml of fresh culture medium. When the cells were 50–70% confluent, cells were passaged repeatedly to remove neurons and enrich the astrocyte population. Typically five passages were required to obtain a pure astrocyte population. Astrocytes were maintained in culture and plated onto 16- or 96-well plates for transport assays.

#### Generation of HEK cell lines stably expressing ASCT1 and ASCT2

The following HEK293 stable cell lines were generated: HEK-ASCT1, HEK-ASCT2. To prepare these cells lines the human SLC1A4 (ASCT1; accession number NM_003038.2) and human SLC1A5 (ASCT2; accession number NM_005628.1) were cloned into the pcDNA3.1 vector (Life Technologies) to enable transfection into the HEK-293 cell lines using Lipofectamine (Life Technologies). Stable cells were established after selection with 100μg/ml of zeocin for 3 weeks. Human ASCT1 or human ASCT2 expression on the cells was confirmed by PCR. The cells were then maintained in growth media (DMEM supplemented with 10% fetal bovine serum, 1% antibiotic-antimycotic, 100 μg/ml zeocin) and plated onto 96-well poly-D-lysine coated plates at a density of 80,000 cells per well one day prior to assay.

### Transport Assays

Plated cells were brought to room temperature. Each row of cells on the 16- or 96-well plate was assayed sequentially as follows: the medium was aspirated and each well was washed twice (1ml for 16-well plates and 0.25ml for 96-well plates) with transport assay buffer (NaCl 150 mM, KCl 2 mM, MgCl_2_ 1 mM, CaCl_2_ 1 mM, HEPES:Tris buffer 10 mM, pH 7.4). 100μl (96-well plates) or 400μl (16-well plates) of transport assay buffer containing radiolabeled substrate (typically at 0.5 μM final concentration) with or without test compounds was added to each well and incubated at room temperature: typically 5 or 20 min for astrocytes and 1 min for HEK cell lines; the incubation was terminated by aspiration of the assay solution and each well washed twice with assay buffer at 4⁰C (1ml for 16-well plates and 0.25ml for 96-well plates). The cells were then solubilized in 1% Triton X-100 for at least 45 min, and an aliquot taken for liquid scintillation counting. For measuring exchange, the same procedures were used, except that astrocytes were loaded with radiolabeled substrate in transport assay buffer at room temperature for 5 min, followed by aspiration and one wash with transport assay buffer. Transport assay buffer (without radiolabeled substrate) containing test amino acids was added immediately and incubation continued typically for 10 min. The supernatant was collected and an aliquot taken for liquid scintillation counting to determine the amount of radiolabeled substrate that had been exchanged into the assay medium.

#### Patch clamp Experiments

HEK cells expressing ASCT1 or ASCT2 were plated on 12 mm coverslip (354086, BD BIOSCIENCES). Before recording, a coverslip was removed from the incubator, and the culture medium was replaced with recording saline (in mM): NaCl 140, CaCl_2_ 2, MgCl_2_ 2, HEPES 10, titrated to pH 7.4 with NaOH. Whole-cell recordings were made at room temperature with pipettes pulled in four stages from 1.5 mm outer diameter glass capillary tubes (WPI, Sarasota, FL) with a P-97 micropipette puller (Sutter Instruments, Novato, CA). Patch pipettes were filled with a solution containing (in mM): NaSCN 130, EGTA 10, MgCl_2_ 2, HEPES 10, and L-Alanine 10, pH 7.3. Ionic currents were measured with patch-clamp amplifiers (Axopatch 200B, Molecular Devices, Sunnyvale, CA), filtered at 2 kHz, digitized at 10 kHz. The cells were voltage-clamped at a holding potential of -60 mV. L- and D-serine, L-glutamine and L-trans-4 hydroxyproline (t-Pro) were applied for 2s through the Octaflow system (ALA Scientific Instruments, Farmingdale, NY), which produced concentration rise times of less than 15 msec. The amplitudes of sustained currents were measured 1700 msec after amino acid application as the mean current over an interval of 200 msec.

#### Oocyte flux assays

Oocytes were injected with approximately 50 ng of human SLC1A4 or SLC1A5 cRNA, and voltage-clamp current recordings or radiolabel flux assays were performed 3–5 days later. Recording electrodes (0.2–1.0 MΩ) were filled with 3M KCl. Recording solution (frog Ringer) contained: 46 mM NaCl, 50mM NaSCN, 2 mM KCl, 1 mM MgCl_2_, 1.8 mM CaCl_2_, 5 mM HEPES pH 7.5 The recording chamber was grounded with a 3 M KCL-filled agar bridge connecting to a 3 M KCl reservoir containing an Ag/AgCl electrode. Oocytes were voltage-clamped with GeneClamp 500 amplifiers and analog-digital converters (Molecular Devices) interfaced to a PC or MacPro. Data were acquired and analyzed with Axograph and Kaleidagraph software. Oocytes expressing transporters or control, un-injected oocytes were incubated with indicated concentrations of [^3^H]-labeled amino acids (American Radiolabeled Chemicals; 20–60 Ci mmol^−1^) in Ringer solution. Uptake was stopped by washing 3 times with 4°C buffer, then oocytes were lysed in 1.0% sodium dodecylsulfate, and radioactivity was measured by liquid scintillation spectroscopy. Homo- or heteroexchange release of [^3^H]D-serine was measured after labeling the internal neutral amino acid pool by incubating oocytes with carrier-free [^3^H]D-serine for 1h followed by transfer into wells containing 500 ml of Ringer’s solution for 60 s in the presence of various test amino acids or control Ringer’s solution as described [32].

### Statistical Analyses

Statistical analyses were performed using a two-tailed Student’s t-test. For comparison of a one-site versus a two-site fit in the astrocyte transport experiments the extra sum-of-squares F-test in GraphPad Prism6 was used (GraphPad, La Jolla, CA). Values were deemed significantly different when p<0.05.

## Results

### Transport studies in astrocyte cultures

**Fig 1:**
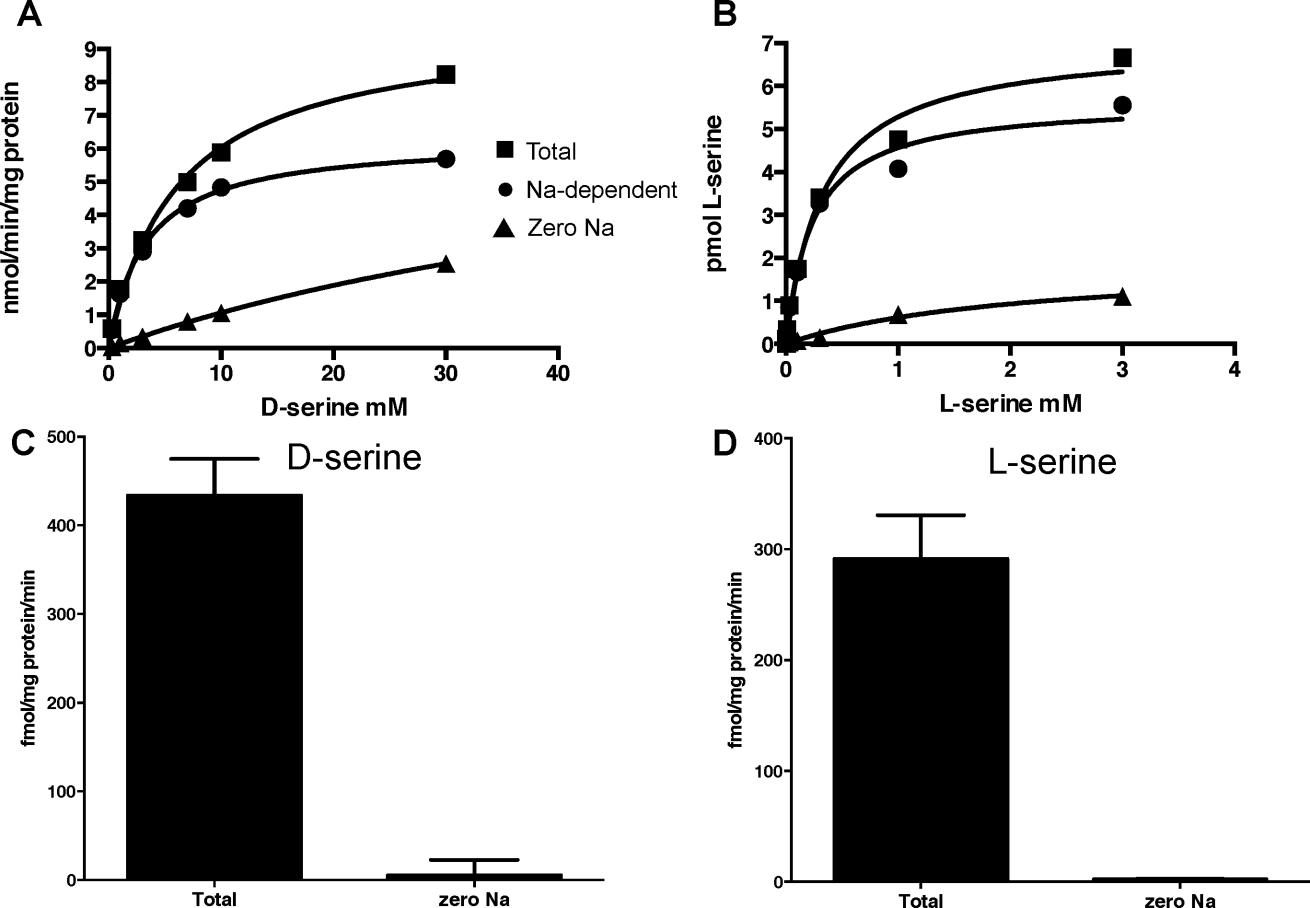
Transport of [^3^H]D-serine and [^3^H]L-serine by rat hippocampal astrocyte cultures. Transport was conducted as described in Materials and Methods. (A,B) Transport of [^3^H]D-serine (A) or [^3^H]L-serine (B) over a range of substrate concentrations in the presence and absence of sodium. Data are from a single experiment that was repeated an additional 2 times for [^3^H]D-serine and 7 times for [^3^H]L-serine. Curves were fitted using GraphPad Prism (see Table 1 for mean K_m_ and V_max_ values). The sodium-dependent transport was calculated by subtracting transport in the presence of sodium from that in buffer where sodium was replaced by equimolar choline. (C,D) Dependence of [^3^H]D-serine (C) or [^3^H]L-serine (D) transport on extracellular sodium. Experiments were conducted in sodium-containing (Total) or choline chloride-containing (zero Na) buffer at a substrate concentration of 0.5 μM. For [^3^H]D-serine transport n = 3, for [^3^H]L-serine transport n = 12.

[^3^H]L- and D-serine were taken up by rat hippocampal astrocyte cultures in a time, temperature and sodium-dependent manner. At low substrate concentrations, transport was almost entirely dependent on the presence of sodium (Fig 1). When sodium was replaced by equimolar choline, residual uptake was approximately 1 percent of uptake in the presence of sodium for both [^3^H]L- and D-serine. The sodium-dependent transport of both serine isomers obeyed Michaelis-Menten kinetics and gave similar V_max_ values consistent with the notion that they are transported by the same system(s) (Fig 1; Table 1). L-serine had a 10-fold higher affinity than D-serine, with K_M_ values of 194 and 2200 μM, respectively (Table 1).

**Table 1:** Kinetic values for [^3^H]L-serine and [^3^H]D-serine transport into rat hippocampal astrocyte cultures.

Substrate	K_M_ *μM*	V_max_ *nmol/min/mg protein*	N
[^3^H]L-serine	194±26	4.42±0.57	8
[^3^H]D-serine	2200±830	5.03±0.80	3

Values are the mean ± SEM of n observations.

**Fig 2:**
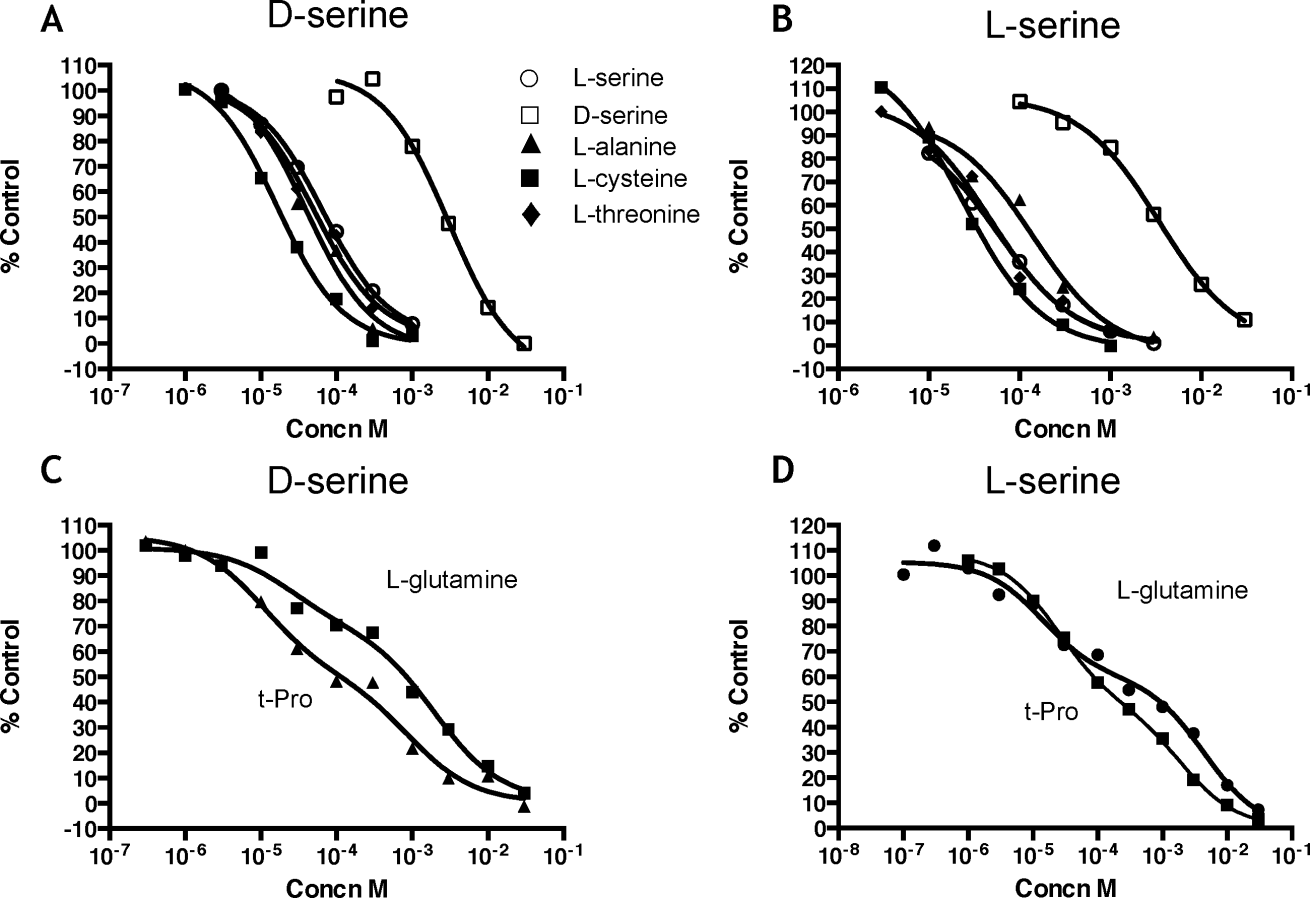
Inhibition by amino acids of [^3^H]D-serine and [^3^H]L-serine transport into rat hippocampal astrocyte cultures. Transport was conducted as described in Materials and Methods. (A,C) Inhibition of [^3^H]D-serine transport by amino acids; (B,D) Inhibition of [^3^H]L-serine transport by amino acids. Values are percent control and are from a single experiment that was repeated 2–7 times. Curves were fitted using GraphPad Prism (see Tables 2 and 3 for mean IC_50_ values) with a one-site fit For (A) and (B) and a two-site fit for (C) and (D).

Transport of both isomers was inhibited by small neutral amino acids, consistent with the reported substrate specificity of system ASCT. L-alanine, L-serine, L-cysteine and L-threonine were all effective inhibitors, with similar IC_50_ values for the transport of both of [^3^H]L-serine and [^3^H]D-serine (Fig 2; Table 2). In contrast, glycine and L-proline were weak inhibitors as was the system A inhibitor, MeAIB, which gave less than 50% inhibition at 10mM when tested against [^3^H]L-serine transport (Table 2).

**Table 2:** IC_50_ values for inhibition by amino acids of [^3^H]L-serine and [^3^H]D-serine transport into rat hippocampal astrocyte cultures.

*Amino acid*	[^3^H]L-serine Transport *IC*_*50*_ *μM*	N	[^3^H]D-serine Transport *IC*_*50*_ μ*M*	N
L-alanine	107.9±22.0	3	60.2±12.3	3
L-threonine	43.2±6.8	3	67.7±51.3	3
L-cysteine	37.2±11.7	3	22.6±4.1	3
L-serine	83.9±9.8	6	60.9±4.9	3
D-serine	2805±380	4	2516±396	8
Glycine	1818±397	3	1626±267	3
L-proline	1866±125	3	2290±23.6	3
MeAIB	>10,000	3	ND	

Values are the mean ± SEM of n observations. MeAIB is N-methylaminoisobutyrate.

Two amino acids exhibited a multiphase inhibition profile. L-glutamine and L-trans-4-hydroxyproline (t-Pro) produced shallow inhibition curves that were best described by a 2-component fit (Fig 2). With [^3^H]D-serine as the substrate, a 2-component fit was significantly better than a 1-component fit for both L-glutamine and t-Pro in 3 out of 3 experiments (P<0.05, t-test). With [^3^H]L-serine as the substrate, inhibition by L-glutamine was best described by a 2-component fit in 7 out of 7 experiments (P<0.05, t-test) and inhibition by t-Pro was best described by a 2-component fit in 7 out of 8 experiments (P<0.05, t-test). The high- and low-affinity components for L-glutamine inhibition represented 35% and 65% of the total transport of [^3^H] D-serine, and 42% and 58% of the total transport of [^3^H] L-serine, respectively (Table 3). For t-Pro, the high- and low-affinity components were 61% and 39% of the total transport of [^3^H] D-serine, and 46% and 54% of the total transport of [^3^H] L-serine, respectively (Table 3). This suggests that two components of L- and D-serine transport may exist: one defined by a high affinity for L-glutamine (and low affinity for t-Pro), and a second with a high affinity for t-Pro (and a low affinity for L-glutamine).

**Table 3:** Values for multiple components of inhibition by L-glutamine and t-Pro of [^3^H]L-serine and [^3^H]D-serine transport into rat hippocampal astrocyte cultures.

**[**^**3**^**H]D-serine Transport**	**Component 1** *IC*_*50*_ *μM*	**Component 2** *IC*_*50*_ *μM*	**Component 1** *(%)*	**N**
L-glutamine	26.0±2.8	2494±320	35.1±0.02	3
t-Pro	23.9±9.4	2869±1463	60.6±0.06	3
**[**^**3**^**H]L-serine Transport**	**Component 1** *IC*_*50*_ *μM*	**Component 2** *IC*_*50*_ *μM*	**Component 1** *(%)*	**N**
L-glutamine	30.3±11.3	3030±691	41.7±4.0	7
t-Pro	21.3±4.9	2498±299	46.0±4.8	8

Components 1 and 2 were defined by the two-site curve-fitting program in GraphPad Prism. Values are the mean ± SEM of n observations. t-Pro is L-trans-4-hydroxyproline.

### Exchange studies in astrocyte cultures

**Fig 3:**
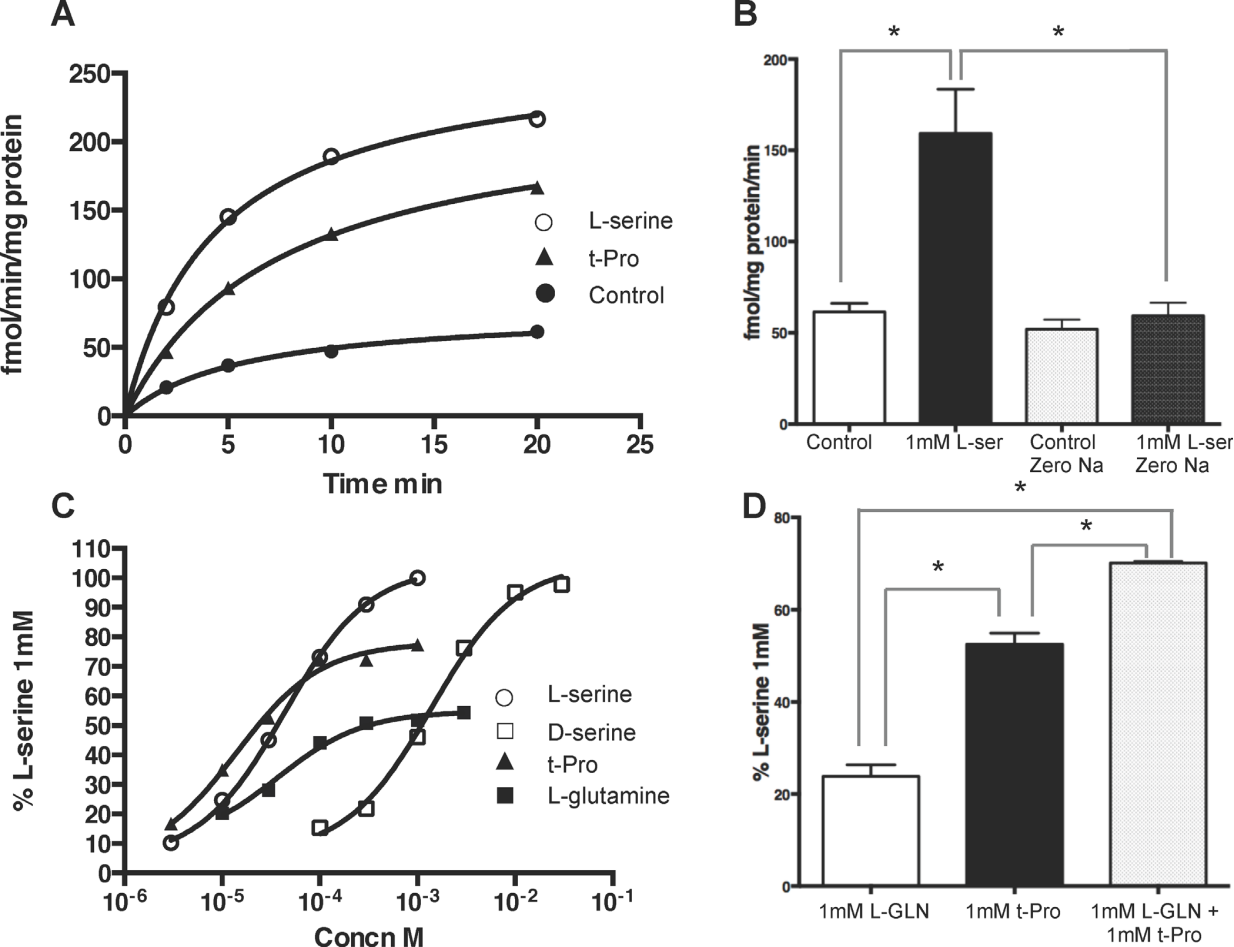
Exchange of [^3^H]L-serine by rat hippocampal astrocyte cultures. After loading of cells with [^3^H]L-serine, amino acids were added to evoke exchange of [^3^H]L-serine into the supernatant as described in Materials and Methods. (A) Time course of exchange under control conditions (no added amino acid) and after addition of 1mM L-serine or 1mM t-Pro. Note the lower maximal effect of t-Pro. Data are from a single experiment that was repeated twice. (B) L-serine-evoked exchange is dependent on sodium; * p<0.01, t-test, n = 7 per group. (C) Concentration-response curves for exchange evoked by L- and D-serine, L-glutamine and t-Pro. Values are expressed as a percentage of the exchange caused by 1mM L-serine after subtraction of exchange in the absence of added amino acids. Curves were fitted using GraphPad Prism with a one-site fit. Data are from a single experiment that was repeated 3–14 times. EC_50_ and maximal effect values are shown in Table 4. (D) Effects of L-glutamine and t-Pro and their combination on exchange. Values are expressed as a percentage of the exchange caused by 1mM L-serine after subtraction of exchange in the absence of added amino acids; * p<0.01, t-test, n = 3 per group.

Since ASCT1 and ASCT2 function by exchange of substrates across the plasma membrane [32], experiments were conducted in the astrocyte cultures to use exchange as a measure of the substrate activity of tested amino acids [27]. Astrocytes were allowed to accumulate [^3^H]L-serine for 5 min under the same conditions used for the transport experiments. The assay buffer was then removed, the cells washed once with assay buffer and then exposed for different times (typically 10 min) to assay buffer containing test amino acids. If the added amino acids are substrates for ASCT1 and ASCT2, they would exchange the accumulated intracellular [^3^H]L-serine into the extracellular medium. The supernatant was then collected to determine the amount of radiolabel that appeared in the assay buffer. Under control conditions with no added amino acid, a small time-dependent accumulation of radiolabel occurred in the supernatant, and in the presence of L-serine (1mM) this was greatly accelerated reaching a maximum at 20 min (Fig 3A). This effect of L-serine was sodium-dependent since replacement of sodium by choline prevented L-serine-induced exchange (Fig 3B); however the exchange observed in the absence of added L-serine was sodium-independent (Fig 3B). As shown in Table 4, amino acids provoked exchange with EC_50_ values that were similar to the IC_50_ values measured in the uptake experiments. All of the known ASCT substrates tested caused exchange, but gave different maximal effects. Relative to L-serine (100%), L-cysteine, L-proline, L-glutamine and t-Pro all gave maximal exchange values (37–63%) that were lower than L-serine (see also Fig 3A). This could mean that these amino acids are substrates that cannot exchange to the same degree or at the same rate as L-serine, L-alanine or L-threonine, or that they selectively exchange through one component of transport (see below). For L-glutamine and t-Pro, amino acids that had shown evidence for two components in the uptake experiments, a limited maximum exchange value might indicate preferential exchange through one of these components. In support of this, the exchange observed at a maximally effective concentration (1mM, see Fig 3C) of L-glutamine combined with t-Pro gave a significantly greater exchange value than either amino acid alone (Fig 3D). This suggests that, similar to the uptake experiments, L-glutamine and t-Pro evoke exchange preferentially through different components. Notably, D-serine gave a maximal value of exchange identical to that of L-serine indicating that it is a fully functional substrate for both components of L-serine transport.

**Table 4:** EC_50_ and maximum effect values for amino acid-evoked exchange of [^3^H]L-serine from rat hippocampal astrocyte cultures.

*Amino acid*	Astrocyte Exchange		
	*EC*_*50*_ *μM*	*Max*. *Effect %*	N
L-alanine	178±77	90±6.4	4
L-threonine	69.1±21.2	88.2±4.4	3
L-cysteine	27.2±10.8	53.5±2.1	4
L-serine	135±16	100	15
D-serine	2139±630	104±6	4
L-proline	5267±1975	41.4±10.4	3
L-glutamine	41.6±5.7	37.3±6.6	6
t-Pro	22.3±4.7	63.2±7.5	4

Values are the mean ± SEM of n observations. t-Pro is L-trans-4-hydroxyproline.

### Transport and patch clamp studies in HEK cells expressing human ASCT1 and ASCT2

**Fig 4:**
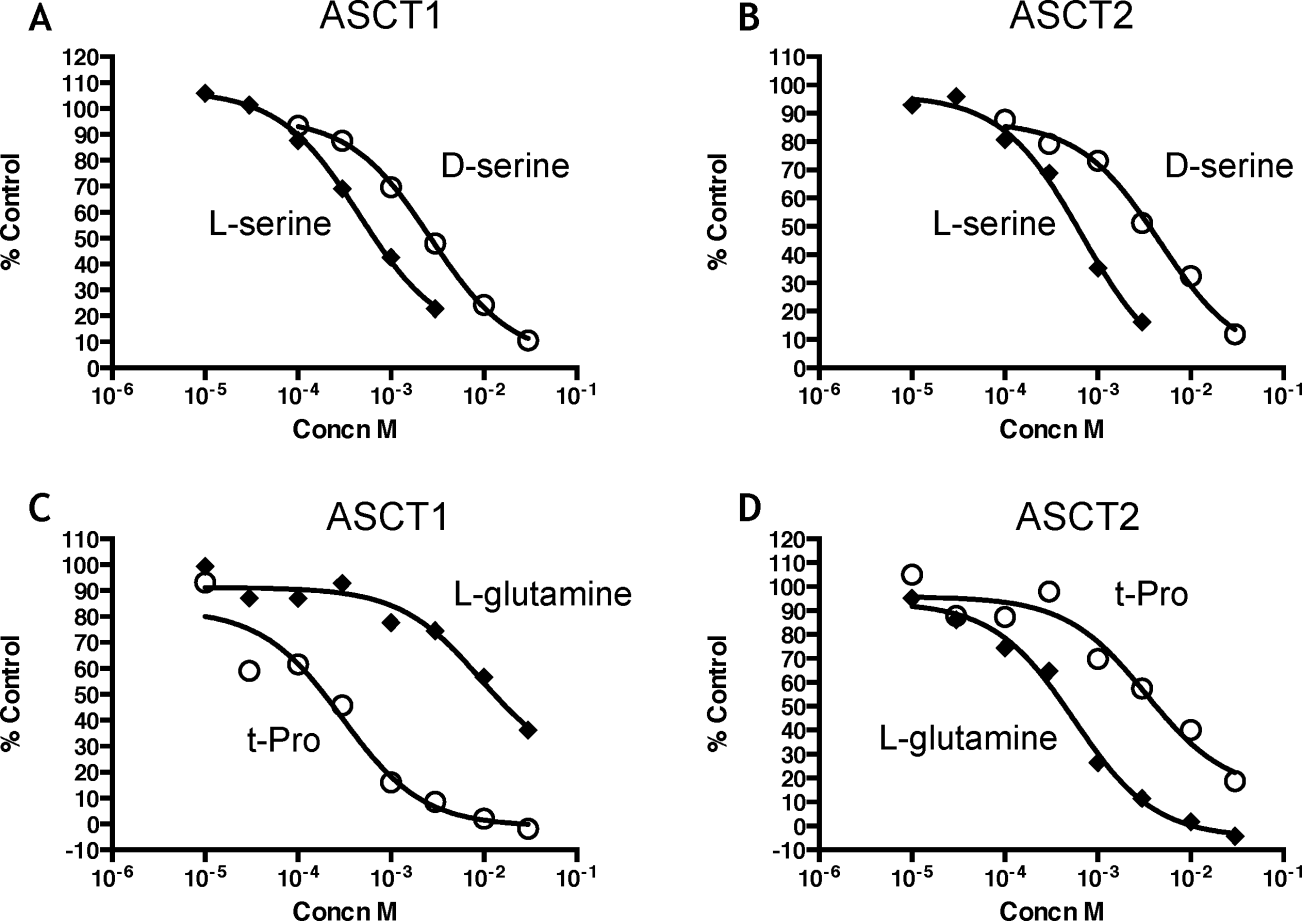
Inhibition by amino acids of [^3^H]L-serine transport into HEK cells expressing human ASCT1 and human ASCT2. Transport was conducted as described in Materials and Methods. (A,C) Inhibition of [^3^H]L-serine transport by amino acids in HEK cells expressing human ASCT1; (B,D) Inhibition of [^3^H]L-serine transport by amino acids in HEK cells expressing human ASCT2. Values are percent control and are from a single experiment that was repeated 2–5 times. Curves were fitted using GraphPad Prism (see Table 5 for mean IC_50_ values) with a one-site fit.

L-glutamine and t-Pro are substrates transported with high apparent affinity by ASCT2 [26] and ASCT1 [33] respectively. This raised the interesting possibility that both ASCT1 and ASCT2 transporters were present in astrocytes and represented the high affinity components of transport observed for t-Pro and L-glutamine, respectively. To explore this further, human ASCT1 or ASCT2 were permanently expressed in HEK cells. Expression of either ASCT1 or ASCT2 increased [^3^H]L-serine transport over the parental HEK cells. Similar to the astrocytes, [^3^H]L-serine transport was almost entirely sodium dependent. In HEK cells expressing ASCT1, control [^3^H]L-serine transport in the presence of sodium was 1266±278 fmol/min/mg protein (n = 3), and in the absence of sodium was 33.4±35.4 fmol/min/mg protein (n = 3). In HEK cells expressing ASCT2, control [^3^H]L-serine transport in the presence of sodium was 1362±257 fmol/min/mg protein (n = 3), and in the absence of sodium was 103±146 fmol/min/mg protein (n = 3). Comparison of inhibition by amino acids revealed that [^3^H]L-serine transport in both cell lines retained an ASCT profile similar to that in astrocytes, although IC_50_ values were 3–10 fold greater than those observed in the astrocyte cultures (Table 5). Notably, D-serine inhibited transport into both ASCT1 and ASCT2-expressing cells (Fig 4A and 4B). In addition, t-Pro was more potent as an inhibitor of [^3^H]L-serine

**Table 5:** IC_50_ values for inhibition by amino acids of [^3^H]L-serine transport into HEK cells expressing human ASCT1 or ASCT2.

*Amino acid*	ASCT1 *IC*_*50*_ *μM*	N	ASCT2 *IC*_*50*_ *μM*	N
L-alanine	328±40	3	365±110	3
L-threonine	181±75	3	228±83	3
L-cysteine	198±58	3	372±84	3
L-serine	289±64	5	704±215	5
D-serine	3937±1907	3	4913±436	3
Glycine	8074±977	3	7431±1231	2
L-proline	2863±883	3	>10,000	3
MeAIB	>20,000	3	>20,000	3
L-glutamine	4581±1989	6	555±66	5
t-Pro	189±10	6	3574±632	6

Values are the mean ± SEM of n observations. MeAIB is N-methylaminoisobutyrate; t-Pro is L-trans-4-hydroxyproline.

Transport in ASCT1-expressing cells than in ASCT2-expressing cells and conversely L-glutamine was more potent as an inhibitor of [^3^H]L-serine transport into ASCT2-expressing than ASCT1 expressing cells (Table 5; Fig 4C and 4D).

**Fig 5:**
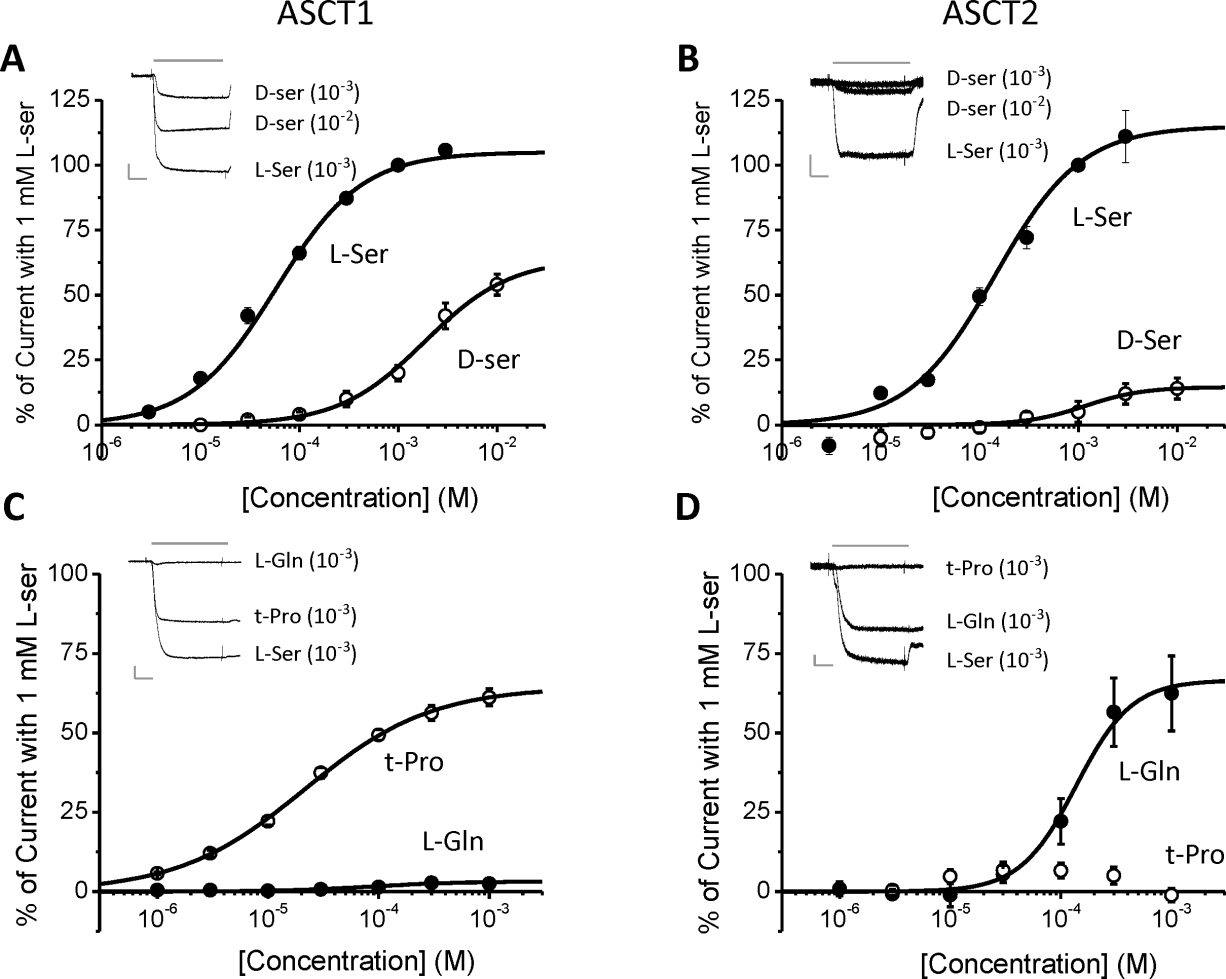
Responses induced by amino acids in patch-clamp recording from HEK cells expressing human ASCT1 and human ASCT2. (A,C) Dose-response of currents activated by amino acids in HEK cells expressing human ASCT1; (B,D) Dose-response of currents activated by amino acids in HEK cells expressing human ASCT2. All currents were normalized to those evoked by 1 mM L-serine (100%). Each data points represent the mean±SEM of 4 to 7 cells. The mean EC_50_ values are shown in Table 6. The inserts are representative currents activated by amino acids at the concentrations shown in parentheses. Grey lines above the current traces indicate application times. The lower scale bars in grey are vertical axis: current amplitude (200 pA for A,C and 50 pA for C,D); horizontal axis: time (500 ms).

Patch clamp recordings from HEK cells expressing human ASCT1 or ASCT2 were used to further characterize these transporters, by measuring the transport-associated anion conductance [34]. For HEK cells expressing either sub-type, L- and D-serine evoked currents with EC_50_ values that were close to those obtained in the radiolabeled substrate transport experiments (Fig 5; Tables 5 and 6). D-serine evoked substrate-like responses in cells expressing either sub-type although the maximal effect was less than that for L-serine, particularly for ASCT2. As previously reported [34], L-glutamine evoked robust currents in ASCT2-expressing cells, but was without effect in ASCT1-expressing cells. t-Pro had the opposite profile. These data further confirm that t-Pro and L-glutamine can be used as selective substrates for ASCT1 and ASCT2, respectively.

**Table 6:** EC_50_ values for amino acid-induced anion currents in HEK cells expressing human ASCT1 or ASCT2.

*Amino acid*	ASCT1 *EC*_*50*_ *μM*	N	ASCT2 *EC*_*50*_ *μM*	N
L-serine	60 ± 8	5	146 ± 20	5
D-serine	1847 ± 182	4	1129 ± 431	4
L-glutamine	>1000	6	169 ± 42	7
t-Pro	21 ± 1	6	>1000	7

Values are the mean ± SEM of n observations. t-Pro is L-trans-4-hydroxyproline.

Taken together, these data support the idea that the two components of transport in the astrocyte cultures represent ASCT1 and ASCT2, with the component blocked by t-Pro with high affinity being mediated by ASCT1 and the component blocked by L-glutamine with high affinity being mediated by ASCT2. It follows that D-serine must be a substrate for both ASCT1 and ASCT2. In the literature, it has been commonly stated that sodium-dependent transport of D-serine is mediated by ASCT2 but not ASCT1 (e.g. [27,35]) based on an earlier study by Shafqat et al [36] reporting that D-serine had no effect on transport mediated by ASCT1 in the *Xenopus* oocyte expression system.

### Transport and voltage clamp studies in *Xenopus* oocytes expressing human ASCT1 and ASCT2

**Fig 6:**
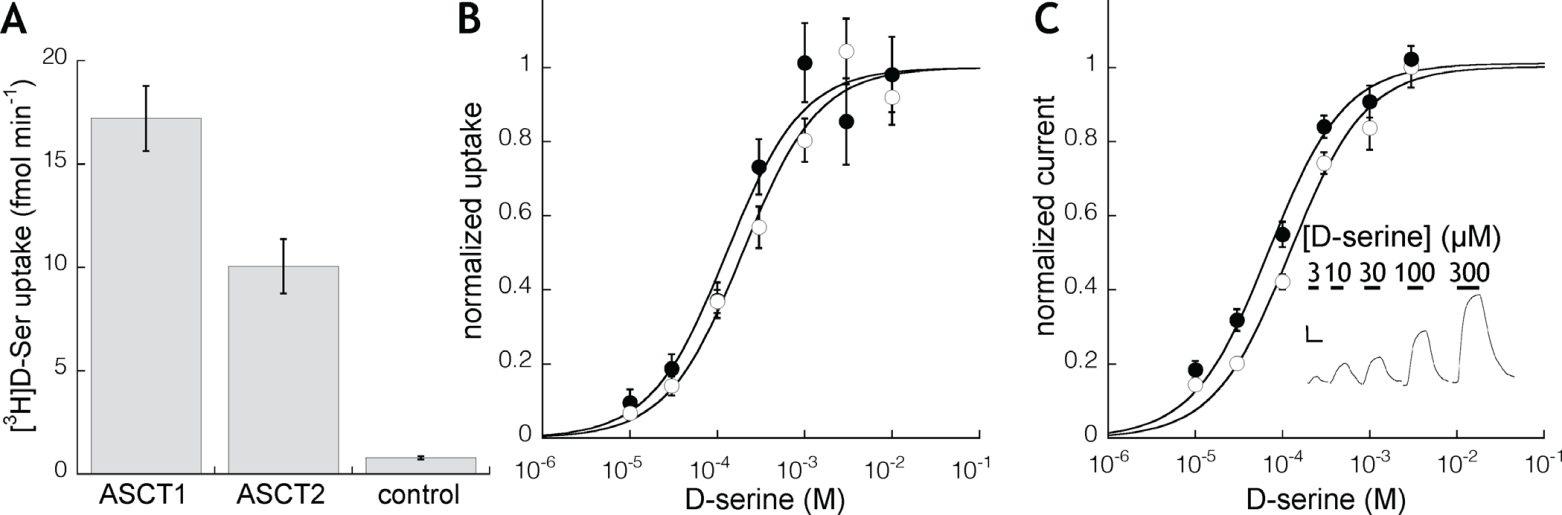
Uptake of D-serine by ASCT1 and ASCT2 expressed in *Xenopus* oocytes. (A) Uptake of 100nM [^3^H]D-serine by Xenopus oocytes injected with human cRNA encoding SLC1A4 (ASCT1; n = 44), SLC1A5 (ASCT2; n = 45), or control, uninjected (n = 30). Uptake was significantly increased by mRNA injection (data from 4 batches of oocytes; bars represent mean ± SEM; p<0.001). (B) Concentration-dependence of [^3^H]D-serine uptake into oocytes injected with cRNA encoding SLC1A4 (filled circles) or SLC1A5 (open circles). Data points (mean ± SEM; n>6) fitted to the Michaelis-Menten function with K_M_ values of 206 and 167 μM for SLC1A4 and SLC1A5, respectively. (C) Concentration-dependence of currents induced by D-serine in voltage-clamped oocytes injected with cRNA encoding SLC1A4/ASCT1 (closed circles; K_M_ = 155+/-22 μM, n = 18) or SLC1A5/ASCT2 (open circles; K_M_ = 107 ± 15 μM, n = 25). Inset shows representative recording in oocyte expressing SLC1A4; D-serine was superfused for durations and concentrations (μM) indicated by bars. Holding potential = -20 mV; scale bars 20 nA/60 sec. D-serine did not induce currents in uninjected oocytes (data not shown).

In order to further investigate the interaction of human ASCT1 and ASCT2 transporters with D-serine, *Xenopus laevis* oocytes were microinjected with cRNAs encoding the transporters and assayed 3–5 days later for the ability to mediate [^3^H]D-serine flux. Following a 10 minute incubation, oocytes expressing ASCT1 or ASCT2 mediated significantly greater uptake of 100 nM [^3^H]D-serine than did control uninjected oocytes (Fig 6A). The uptake of [^3^H]D-serine mediated by each transporter was concentration-dependent and saturable. The K_M_ values estimated by fitting normalized concentration-response curves from six groups of oocytes was 206 and 167 μM for SLC1A4 and SLC1A5, respectively (Fig 6B). Voltage clamp experiments in oocytes expressing ASCT1 or ASCT2 revealed D-serine-evoked currents for both sub-types that displayed properties consistent with the anion-selective conductance activated by other neutral amino acid substrates [37]. In addition, the D-serine-evoked anion currents displayed concentration-response curves and K_M_ values similar to those derived from the radiolabel uptake experiments (Fig 6C).

**Fig 7:**
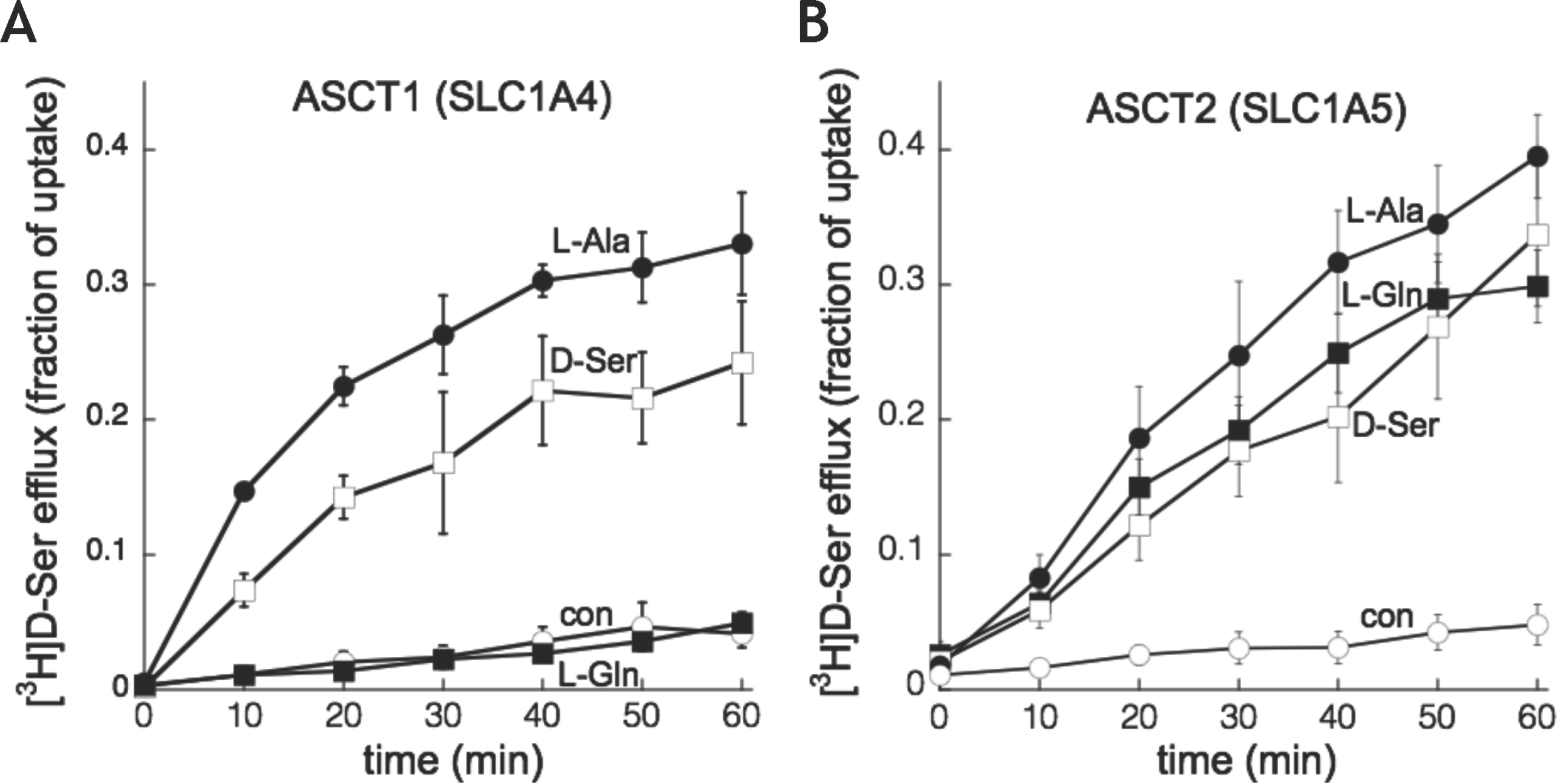
**Homo- and hetero-exchange of intracellular [**^**3**^**H]D-serine with extracellular test substrates in oocytes injected with human cRNA encoding SLC1A4 (A) or SLC1A5 (B).** Ooctytes were pre-incubated with 100nM [^3^H]D-serine for 60 min, washed, and then incubated in Ringer containing 1mM indicated unlabeled amino acid. Extracellular radioactivity counted at indicate times is expressed as the mean fraction of total intracellular [^3^H]D-serine. Points represent mean ± SEM of 4 experiments with 5–6 oocytes per substrate tested. The amino acid L-glutamine is selectively transported by SLC1A5 over SLC1A4 and displayed similar selectivity for heteroexchange with D-serine.

Both ASCT1 and ASCT2 have been shown to mediate uptake by a heteroexchange mechanism [37,38]. The bidirectional and reversible nature of exchange means that either transporter could serve to mediate efflux and/or influx of D-serine, depending on the transmembrane gradients of the respective amino acid substrate pools and local intracellular and extracellular concentrations of D-serine. We characterized efflux of intracellular D-serine by preloading oocytes expressing ASCT1 or ASCT2 with 100 nM [^3^H]D-serine for 60 min and then measuring release of radioactivity in response to addition of extracellular (non-radioactive) substrate. Both transporters mediated robust trans-substrate stimulation of efflux in a manner consistent with each transporter’s substrate selectivity. In particular, ASCT2 mediated release of D-serine in response to the presence of trans L-glutamine, while ASCT1-expressing cells did not (Fig 7).

## Discussion

The importance of D-serine as an endogenous regulator of NMDA receptor function has driven investigations of the cellular mechanisms that control extracellular D-serine levels in the CNS [24,35,39,40]. However, significant gaps in understanding remain, and there is a pressing need to identify and characterize all of the mechanisms that contribute to D-serine homeostasis in brain. Although an early report suggested that D-serine was not significantly transported by ASCT1 [36], recent work indicating that HEK cells transfected with either ASCT1 or ASCT2 exhibited increased uptake of D-serine [24] prompted us to quantitatively characterize the kinetics of D-serine flux mediated by these transporters in exogenous systems. The present data demonstrate for the first time that D-serine is indeed a substrate for the neutral amino acid transporter ASCT1 with physiologically relevant kinetic parameters. Both transporters also mediated heteroexchange of intracellular D-serine for extracellular neutral amino acid substrates. In addition, both ASCT1 and ASCT2 transporter subtypes were found to mediate D-serine flux in astrocytes cultured from rat hippocampus.

Previous studies have described both L- and D-serine transport in cultured astrocytes [27,29,41,42]. Similar to our findings, D-serine transport was shown to be almost entirely sodium dependent (at relatively low substrate concentrations), and to have the characteristics ascribed to the small neutral amino acid system, ASCT2. These previous studies concluded that astrocytic D-serine transport was mediated primarily by ASCT2, since prior studies in the *Xenopus* oocyte expression system indicated that D-serine is a substrate for ASCT2 [23] but not ASCT1 [36]. The present findings are in general agreement with these previous studies in terms of the sodium dependence and substrate specificity of uptake, but they contradict previous conclusions regarding D-serine transport mediated by ASCT1. We have uncovered two components of D-serine and L-serine transport that are consistent with the presence of both ASCT1 and ASCT2 in astrocyte cultures. In particular, the amino acids L-glutamine and t-Pro show two-component inhibition curves using either D-serine or L-serine as the substrate. Based on the proportion of transport for each component, the low-affinity component for L-glutamine (ASCT1) appeared to correspond with the high affinity component for t-Pro and vice versa. Previous studies have shown that L-glutamine has micromolar affinity as a substrate for ASCT2 [23,26] and one study described t-Pro as a substrate with micromolar affinity for ASCT1 [33]. However, no evaluation of the relative affinities of L-glutamine and t-Pro for ASCT1 and ASCT2 has previously been described. Expression of human ASCT1 and ASCT2 in HEK cells led to an increased transport of L-serine that was sodium-dependent and inhibited by amino acid substrates with a similar rank order of IC_50_ values to that observed for D- and L-serine transport in astrocyte cultures, but with lower apparent affinity (see below). Importantly, L-glutamine inhibited transport with a higher affinity in cells expressing ASCT2 than those expressing ASCT1 and, conversely, t-Pro inhibited transport with a higher affinity for cells expressing ASCT1 than those expressing ASCT2. This selectivity was also observed when measuring anion currents evoked by t-Pro or L-glutamine in ASCT1 or ASCT2-expressing HEK cells, respectively. This is also consistent with the ability of trans L-glutamine to stimulate efflux of D-serine from oocytes expressing ASCT2 but not ASCT1 (Fig 7). Together these data confirm the selectivity of ASCT2 for L-glutamine and ASCT1 for t-Pro, and suggest that L-glutamine and t-Pro appear to be useful tools to define ASCT1 and ASCT2 in the context of sodium-dependent neutral amino acid transport. In support of our data indicating transport mediated by both ASCT1 and ASCT2 in astrocytes, Yamamoto et al, 2003, 2004 [41,42] demonstrated the presence of both ASCT1 and ASCT2 in rat telencephalon astrocyte cultures by PCR.

In addition to oocytes expressing ASCT1 and ASCT2, our data with astrocyte cultures demonstrates exchange of accumulated [^3^H]L-serine by extracellular application of ASCT substrates consistent with their postulated roles as obligate exchangers (37). The EC_50_ values for exchange of the amino acids tested correspond well with the IC_50_ values from uptake experiments, indicating that the same transport systems were involved. As was observed for uptake, the exchange was entirely sodium-dependent, although baseline release in the absence of added substrate was sodium-independent. L-glutamine and t-Pro gave maximal values for exchange that were lower than that for L-serine and when applied together at a maximal concentration, gave significantly greater exchange than L-glutamine or t-Pro alone. This is consistent with the two components observed in the uptake experiments that prefer L-glutamine and t-Pro and further support the idea that they represent separate transport components. However, two other substrates that did not distinguish two transport components in astrocytes also gave less than maximal exchange values (L-proline and L-cysteine). In the case of L-proline, data from transport mediated by human ASCT1 and ASCT2 expressed in HEK cells indicated that, like its analog t-Pro, it prefers ASCT1. However, L-cysteine has a similar affinity for both ASCT1 and ASCT2 (Table 5). Consequently, it may be that some substrates, like L-cysteine, may show less than maximal exchange due to other properties such as slower permeation and thus may appear as “partial” substrates.

Anion currents measured in both ASCT1 and ASCT2 cell lines indicate that D-serine was able to evoke currents that are typical of substrates for both subtypes, although with a lower maximal response than L-serine. However, in the uptake and exchange experiments in astrocytes and oocytes, maximal responses to D-serine were typical of other substrates. This is consistent with previous conclusions regarding the stoichiometric uncoupling between transport and anion conductance in this transporter family [37]. Overall, the data with ASCT1 and ASCT2 expressed natively in astrocytes, and heterologously in HEK cells and oocytes, indicate that D-serine is a substrate for both sub-types. Consequently, ASCT1 should be considered as a potentially important component of extracellular D-serine regulation.

The discrepancy between kinetic parameters for D-serine transport between rat astrocyte cultures, HEK cells, and *Xenopus* oocytes expressing ASCT1 and ASCT2 might suggest a species difference. However, variations in apparent affinity of amino acids for human ASCT1 and ASCT2 may be a result of heterologous expression in systems at different densities and density-to-cell-volume ratios. Previous data with the related transporter EAAT3 (SLC1A1) indicated that transporter surface density correlated inversely with apparent substrate affinity as a consequence of diffusional concentration gradients [43]. It is likely that, compared with the rat astrocyte cultures, permanent expression of human ASCT1 and ASCT2 achieves high cellular transporter levels in the HEK cells. Consequently, differences between the apparent affinities are more likely due to the differences in assay systems than true species differences.

In conclusion, our data suggest the possibility that ASCT1/SLC1A4 represents a potentially important mechanism contributing to D-serine homeostasis in brain. Recently, exome analyses from several groups have linked mutations in SLC1A4 (ASCT1) with syndromes including cognitive impairment, microcephaly, and developmental delay [44–46]. One of the mutations, E256K, has a carrier frequency of 0.7% in the Ashkenazi-Jewish population studied, and results in an increase in apparent affinity and a decrease in maximal velocity for L-serine [46]. ASCT1 is strongly expressed in human cortical and subcortical brain regions, further highlighting this potential functional link [47]. Further work will be required to explore the contribution of ASCT1 to D-serine homeostasis and to evaluate the possibility that changes in D-serine homeostasis may contribute to the neurological sequelae associated with mutations in this transporter.
